# Bacterial Communities in Lanna Fermented Soybeans from Three Different Ethnolinguistic Groups in Northern Thailand

**DOI:** 10.3390/microorganisms11030649

**Published:** 2023-03-03

**Authors:** Rujipas Yongsawas, Ammarin In-on, Angkana Inta, Jatupol Kampuansai, Hataichanok Pandith, Nakarin Suwannarach, Saisamorn Lumyong, Thararat Chitov, Terd Disayathanoowat

**Affiliations:** 1 Department of Biology, Faculty of Science, Chiang Mai University, Chiang Mai 50200, Thailand; 2 Doctor of Philosophy Program in Applied Microbiology (International Program) in Faculty of Science, Chiang Mai University, Chiang Mai 50200, Thailand; 3 Research Center of Microbial Diversity and Sustainable Utilization, Faculty of Science, Chiang Mai University, Chiang Mai 50200, Thailand; 4 Academy of Science, The Royal Society of Thailand, Bangkok 10300, Thailand

**Keywords:** bacterial diversity, ethnic food, fermented soybean, Thua Nao, Lanna culture, metagenomics

## Abstract

Northern Thailand, the main part of the Lanna region, is home to a diverse range of ethnic groups, each with their own food and cultural heritage. The bacterial compositions in fermented soybean (FSB) products indigenous to three Lanna ethnolinguistic groups, including Karen, Lawa, and Shan, were investigated in this study. Bacterial DNA was extracted from the FSB samples and subjected to 16S rRNA gene sequencing using the Illumina sequencing platform. Metagenomic data showed that the predominant bacteria in all FSBs were members of the genus *Bacillus* (49.5–86.8%), and the Lawa FSB had the greatest bacterial diversity. The presence of genera *Ignatzschineria*, *Yaniella*, *Atopostipes* in the Karen and Lawa FSBs and *Proteus* in the Shan FSB might be indicators of food hygiene problems during processing. The network analysis predicted antagonistic effects of *Bacillus* against some indicator and pathogenic bacteria. The functional prediction revealed some potential functional properties of these FSBs. The presence of *Bacillus* in all FSBs and *Vagococcus* in the Shan FSB suggests that these FSBs could potentially be good sources of beneficial bacteria, and they should be conserved and promoted for health and food security reasons. However, food processing hygiene measures should be introduced and monitored to warrant their properties as health foods.

## 1. Introduction

Northern Thailand, the main part of the ancient “Lanna” region, is an area in which many ethnic minorities have settled. Many ethnolinguistic groups (people classified by language family) live in this area, including the Kra-Dai (the largest group), Sino-Tibetan, Austroasiatic, and Hmong-Mien-speaking families. Furthermore, the cultures of two Asian civilisation groups, the Sinosphere and Indosphere, have spread to this region.

This region has mountainous areas that keep it separate from the surrounding regions. Due to their geographical conditions and history, many Lanna territories are inhabited by different ethnic groups, resulting in diversity of the regional cultures. The culture of an ethnic group is also influenced by the culture of other ethnic groups within the region, which, when combined with the local culture, form a unique ethnic regional group culture. Although the world has entered the modern era, the traditional cultures of the Lanna ethnic groups, including their indigenous food cultures, are still well preserved. Lanna food culture is different from that of other regions of Thailand and the Southeast Asian lowlands.

One of the most popular indigenous foods of the Lanna culture is alkaline-fermented soybean (FSB). Products of this type, indigenous to the different ethnic groups living in Northern Thailand, are known by different names, such as Thua Nao (Shan), Tor-Nor-Au (Karen), and Kuem (Lawa). They have unique flavours and a longer shelf life than cooked soybeans, and they can be well preserved in Northern Thailand’s tropical climate.

Fermented soybeans are made of soybean [*Glycine max* (L.) Merrill] fermented in an alkaline fermentation process which involves the fermentation of protein-rich substances to produce an alkaline condition [1]. Alkaline-fermented foods are usually considered harmless and are found in traditional diets around the world, particularly in Asian and African cultures [2]. In India, Nepal, and Bhutan, kinema is known as one of the oldest foods [3]. In Japan, the best-known product of this type is natto, which has been consumed for thousands of years [4].

A general FSB fermentation process begins with the steps of washing and soaking soybeans overnight, followed by boiling them for two to three hours until they are soft. After that, the cooked soybeans are fermented in a fermenting container. The difference between kinema fermentation and natto fermentation is that kinema soybeans are slightly crushed in a mortar after boiling to increase the surface area for bacteria to ferment more rapidly [5]. A small amount of ash is mixed into the soybeans for kinema fermentation. Then, fresh ferns, or fig plants and banana leaves are placed in bamboo baskets, in which cooked soybeans are placed for fermentation with natural bacteria [6]. For natto fermentation, steamed soybeans are packed in straw bags. Natural bacteria in rice straw create the fermentation process [7]. Straw bacteria also play a part in the fermentation process of cheonggukjang (a fermented soybean product in Korea) [8]. In Thailand, FSB products are fermented foods that are mainly produced and consumed by ethnic people in Northern Thailand. Thai FSB products are made of soybeans (*Glycine max*) [2] that are naturally fermented by *B. subtilis* [2,9,10,11]. Fermented soybean products are available in a variety of forms, from fresh FSB in banana leaf wrapping to dried, mashed FSB sheets (known in Northern Thailand as “Thua Nao Kab”), which can be kept longer than fresh FSB products. Furthermore, different ethnic groups have different FSB manufacturing techniques, resulting in FSB products with different flavours and textures.

Indigenous fermented foods have been known to potentially possess functional properties, which are partly contributed by the microorganisms involved in fermentation. The fermentation of FSB products, such as kinema in India, natto in Japan, cheonggukjang in Korea, and Thua Nao varieties in Thailand, often involves the natural fermentation process of the *Bacillus* species, especially *B. subtilis*, or its relatives [2,9,10,11]. This fermentation process not only gives the FSB products their umami flavour, but also increases the amounts of active compounds in the beans such as volatile compounds, free amino acids, and essential amino acids [12,13], making the FSB products more flavourful and more beneficial than their raw source materials. Not only are there nutritional benefits from FSB products, but there are also benefits from the microorganisms related to the fermentation process. A probiotic *Bacillus*, according to a recent study, can inhibit *Staphylococcus aureus* quorum sensing, causing *S. aureus* to be absent from faecal samples in its presence [14]. Additionally, the functional properties of some indigenous fermented soybean products, especially natto from Japan, have long been recognised. However, there is very limited knowledge on the functions or the microorganisms that play a role in fermentation and the functions of the FSB products of the Shan, Karen, and Lawa of Northern Thailand or the Lanna region, even though they are among the major ethnic groups of the north-western part of Southeast Asia.

In this study, therefore, we will explore bacterial communities in some ethnic FSB products, which are among the fermented food heritages of Lanna, with a particular focus on those in three major ethnolinguistic groups: Karen (a Sino-Tibetan speaking family), Lawa (an Austroasiatic speaking family), and Shan (a Kra-Dai speaking family). The results of this study are expected to advance the knowledge of ethnomicrobiology. This will be useful not only in the understanding and management of nutrition and public health for ethnic groups, but also in food sustainability, the preservation of ethnic food heritages, and the beneficial use of these heritage foods in the modern world.

## 2. Materials and Methods

### 2.1. Fermented Soybean Sampling Sites

FSB samples from three ethnic groups were collected from districts in Mae Hong Son Province, which is located in northern Thailand’s north-western region. These included (1) Karen FSB (Tor-Nor-Au) samples, collected from Ban Mueang Pam village, Pang Mapha district; (2) Lawa FSB (Kuem) samples, collected from Mae La Luang village, Mae La Noi district; and (3) Shan FSB (Thua Nao) samples, collected from Khun Yuam Saturday Market, Khun Yuam district. In total, six samples of FSBs from Karen, Lawa, and Shan were randomly collected.

### 2.2. Fermented Soybean Samples

**Figure 1:**
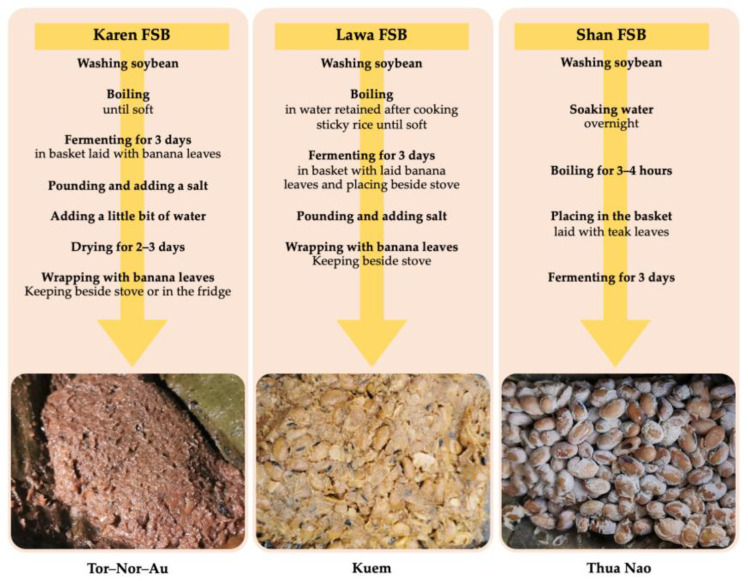
The appearance and production processes of fermented soybean products of three ethnic groups: Karen, Lawa, and Shan, which are known by their native languages as Tor-Nor-Au, Kuem, and Thua Nao, respectively.

The FSB samples from the three ethnic groups were produced using three main steps, including: (1) washing of soybeans; (2) boiling; and (3) fermenting. However, each type of FSB had its own unique details for its production process. In the Shan FSB processing, the pre-fermentation process involved soaking soybeans in water overnight before boiling and fermenting, whereas in the processing of FSBs from the Karen and Lawa, the soaking step was omitted. The soybeans for the Lawa FSB processing were boiled in water retained from sticky rice cooking, whereas the beans for the other FSB processing were boiled only in water. Moreover, the types of plant leaves used during the FSB fermentation and the post-fermentation processes were also different. The production processes of the FSBs are shown in Figure 1.

The FSB products are known by the names Tor-Nor-Au, Kuem, and Thua Nao in the native Karen, Lawa, and Shan languages, respectively. However, all ethnic alkaline soybean products are generally referred to as “Thua Nao” in Thai, as the Shan FSB is in the Shan language.

#### 2.2.1. Analysis of Nutritional Compositions and Physicochemical Properties of Fermented Soybean Products

A one-kilogram portion of the FSB sample from each ethnic group was sent for analysis of the nutritional compositions, including energy, energy from fat, fat, protein, carbohydrate, fiber, sugar, sodium, iron, calcium, potassium, ash, moisture, pH, and water activity. The analysis was performed by the Central Laboratory of the Science and Technology Park, Chiang Mai University, Chiang Mai, Thailand.

#### 2.2.2. Fermented Soybean Sample Preservation

For metagenomic analysis, a one-gram portion of each FSB sample from each ethnic group was drawn and placed in a centrifuge tube. A DNA/RNA Shield (3 mL, Zymo Research, Irvine, CA, USA) was added to each sample. The sample was mixed and kept at −20 °C until DNA extraction was performed.

### 2.3. Bacterial DNA Extraction

The sample from Section 2.2.1 was vigorously mixed using a vortex mixer, then it was left to stand to allow the suspension to be separated from the food material. The suspension was centrifuged at 18,000× *g* for 30 min at 4 °C, and the supernatant was removed. The pellet was used for DNA extraction using the ZymoBIOMICS^TM^ DNA Miniprep Kit (Zymo Research, Irvine, CA, USA).

### 2.4. Bacterial DNA Sequencing

The DNA extracted from each FSB sample (30 µL, with a concentration of 2 ng/µL and an A260/280 value of 1.7 or greater) was subjected to the sequencing of 16S rRNA (V3–V4 regions) using the Illumina platform with the Illumina MiSeq model. The sequencing was performed by Macrogen (Macrogen Inc., Seoul, Republic of Korea). Bacterial metagenomic sequences of the FSB samples were deposited in the NCBI database (accession no. PRJNA747069).

### 2.5. Sequences Processing and Analysis

Bacterial communities in the FSB samples were analysed from the DNA sequences using Quantitative Insights Into Microbial Ecology 2 (Qiime2) software [15]. Sequences were trimmed to remove the primer sequences from the 16S rRNA fragment (Bakt_341F: 5′-CCTACGGGNGGCWGCAG-3′ and Bakt_805R: 5′-GACTACHVGGGTATCTAATCC-3′) [16]. The trimmed sequences were subjected to DADA2 [17] to generate the amplicon sequence variants (ASVs). The singletons were removed from the ASV sequences, and the remaining ASVs were used to generate the rarefaction curve and classify bacterial taxa with SILVA database version 138.1 [18]. The bacterial taxa were plotted in a stack bar format to show the proportions of bacteria found in the FSBs.

### 2.6. Alpha and Beta Diversity Analysis

The singleton-removed ASVs were used to analyse the alpha diversity using PAST software version 4.03 [19] and plotted as an alpha diversity boxplot with a Shannon index. For beta diversity analysis, a one-way PERMANOVA with the Bray–Curtis similarity index was used for the analysis of the significant differences in bacterial diversity among the FSBs from the three ethnic groups. The similarities were plotted in non-metric multidimensional scaling (NMDS), using PAST software version 4.03.

### 2.7. Network Analysis of Bacteria in FSB Bacterial Communities

The correlation network among bacteria in the communities, based on bacterial taxa found in bacterial communities, was analysed using R and RStudio with the “vegan” and “Hmisc” packages. The correlations were generated for a Fruchterman–Reingold plot using Gephi 0.9.2 [20].

### 2.8. Functional Prediction Analysis

Functional genes that could be associated with the bacterial taxa in the FSB samples were predicted by phylogenetic investigation of communities using reconstructing unobserved states 2 (PICRUSt2) software [21] and the ENZYME nomenclature database [22]. The relative abundances of the functional gene activities were demonstrated as a heatmap plot using R [23] and RStudio [23,24].

## 3. Results

### 3.1. Number of Sequences Used in Analysis and Rarefaction Analysis

**Figure 2:**
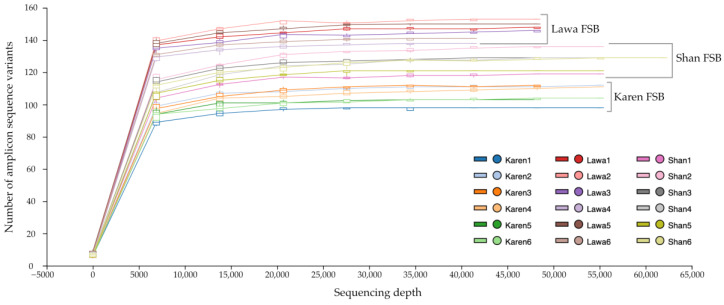
A rarefaction curve plot between bacterial sequencing depth and amplicon sequence variants (ASVs). The bacterial diversity of the Lawa FSB was greatest among the FSBs from the three ethnic groups, as demonstrated by the highest ASVs.

In total, 1,671,516 sequences were obtained from Illumina MiSeq sequencing of the bacterial 16S rRNA gene from all FSB samples. After low-quality sequence removing and denoising, the remaining 986,984 sequences of 474 ASVs were subjected to alpha and beta diversity analyses. The rarefaction curve showed that the ASVs were stable at high sequencing depths. The rarefaction lines of Lawa FSB showed the highest bacterial diversity among the three ethnic FSB varieties. The taxonomic classification was carried out using 99% identity reads with a sequence depth frequency of 43,103, which was the minimum value among all samples (Figure 2).

### 3.2. Bacterial Communities in Fermented Soybeans from Three Ethnic Groups

**Figure 3:**
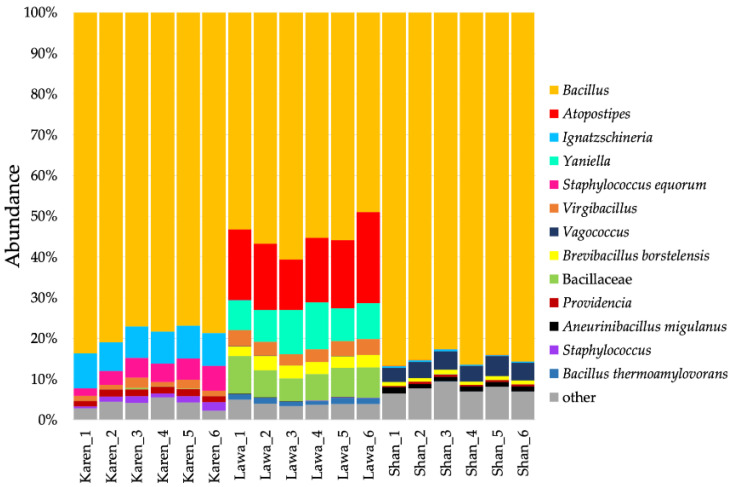
Bacterial proportions in fermented soybean (FSB) products from three ethnic groups: Karen, Lawa, and Shan. The major bacterial taxon found in the FSBs of all ethnic groups was *Bacillus*. Other minor taxa found included *Ignatzschineria*, *Staphylococcus*, and *Virgibacillus* (in the Karen FSB); *Atopostipes*, *Yaniella*, *Bacillaceae*, *Virgibacillus*, and *Brevibacillus* (in the Lawa FSB); and *Vagococcus*, *Proteus*, and *Brevibacillus* (in the Shan FSB).

The major bacterial genus found in all FSB samples from the three ethnic groups was *Bacillus*, which contributed to 76.9–83.6%, 49.0–60.6%, and 82.7–86.8% of the FSB of the Karen, Lawa, and Shan, respectively. However, the minor bacterial components were unique for the FSB from each ethnic group. For example, in the Karen FSB, *Ignatzschineria* (7.1–8.6%) and *Staphylococcus* (2.5–8.3%) were among the unique genera. *Virgibacillus* (1.1–2.5%) was another major group present but found in smaller proportions. In the Lawa FSB, the bacterial community had some unique genera, such as *Atopostipes* (12.3–22.3%), *Yaniella* (7.2–11.5%), and *Brevibacillus* (2.3–3.5%), whereas *Vagococcus* (3.7–5.3%), and *Proteus* (2.8–5.0%) were unique to the Shan FSB. (Figure 2). Analysis at the species level identified some *Staphylococcus* present in the Karen FSB as *S. equorum* and some *Bacillus* in the Lawa FSB as *B. thermoamylovorans* (Figure 3).

### 3.3. Alpha and Beta Diversity

**Figure 4:**
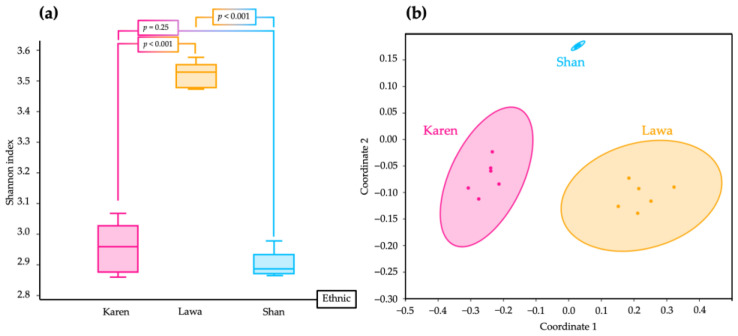
Boxplot of alpha diversity indicated by the Shannon index, which shows some similarities in bacterial diversity between the FSBs of the Karen and the Shan (*p* = 0.25), but differences in bacterial diversity between those of the Karen and the Lawa (*p* < 0.001), and between those of the Lawa and the Shan (*p* < 0.001). Bacterial diversity in the Lawa FSB was greatest among the FSBs from the three ethnic groups (**a**). The NMDS plot shows the differences in bacterial taxa and quantity among the FSBs of the three ethnic groups with separated circles of 95% ellipses (**b**).

From the Shannon index, the alpha diversity of bacteria in the Lawa FSB was highest. It was also different from that of the Karen FSB and the Shan FSB (*p* < 0.001). The alpha diversity of bacteria in the Karen FSB was not significantly different from that of the Shan FSB (*p* = 0.25) (Figure 4a).

The beta diversity analysis showed some differences in bacterial communities between the Karen FSB and the Lawa FSB (*p* = 0.0075), the Karen FSB and the Shan FSB (*p* = 0.0081), and the Lawa FSB and the Shan FSB (*p* = 0.006). There were also differences in the bacterial compositions among the three FSBs based on a one-way PERMANOVA, as shown in the NMDS plot with non-overlap of the 95% ellipse circle (Figure 4b).

### 3.4. Network Analysis

**Figure 5:**
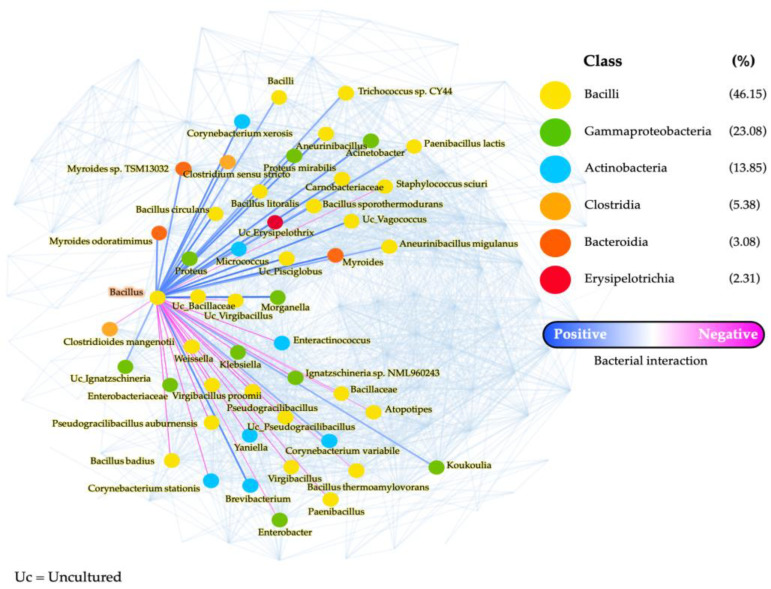
Part of the bacterial correlation network analysis, showing positive and negative correlations between *Bacillus* and another 27 bacterial taxa belonging to six classes, which were present in the fermented soybean products of the Karen, Lawa, and Shan.

Potential interactions among the bacterial taxa found in the FSBs of the Karen, Lawa, and Shan were analysed through correlation network analysis. The overall network analysis plot presents 130 bacterial identities and 2140 interactions among them. There were positive and negative interactions among the bacterial taxa within six major communities (Figure S1; the full version of the network analysis). *Bacillus*, the major bacterial taxon found in every FSB, was potentially negatively correlated with *Ignatzschineria* found in the Karen FSB, and with *Atopostipes* and *Yaniella* found in the Lawa FSB (Figure 5). There were 20 negative interactions and 27 positive interactions detected between *Bacillus* and other taxa belonging to six major bacterial classes, including Bacilli, Gammaproteobacteria, Actonobacteria, Clostridia, Bacteroidia, and Erysipilotrichia (Figure 5).

### 3.5. Nutritional and Physicochemical Properties

Analysis of the FSB nutritional compositions revealed that the Lawa FSB contained more nutrients and minerals than the other two FSBs. The nutritional composition of the Lawa FSB included fat (8.67 g/100 g), protein (19.23 g/100 g), carbohydrate (9.68 g/100 g), fibre (12.69 g/100 g), sugar (0.52 g/100 g), sodium (65.67 mg/100 g), calcium (4.6 mg/100 g), and potassium (54.37 mg/100 g) (Table 1). Although it had a lower total energy content than the other FSBs, it had the highest proportion of energy from fat when compared to total energy (66.47% compared to 52.36% in the Karen FSB and 45.20% in the Shan FSB).

### 3.6. Functional Gene Prediction

**Figure 6:**
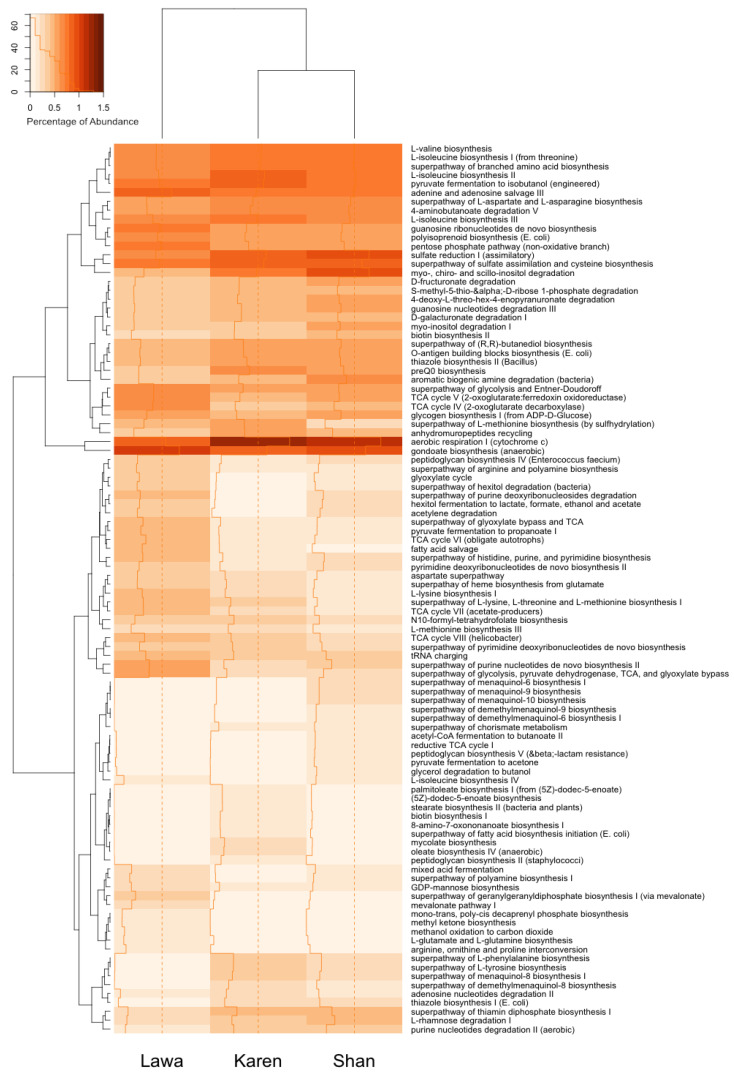
Top 100 functional genes predicted from the bacterial communities in the FSBs from the three ethnic groups, Lawa, Karen, and Shan.

A functional gene heatmap was constructed to demonstrate the metabolic pathways predicted from the bacterial communities in the FSBs of the three ethnic groups. The abundances of the functional pathways predicted for the Karen FSB and the Shan FSB were closer to one another than to those predicted for the Lawa FSB (Figure 6).

## 4. Discussion

Most alkaline FSBs are made of soybeans fermented with naturally occurring microorganisms. The fermentation process helps preserve this type of proteinaceous food. Alkaline-fermented soybean products are found in many parts of the world and can come in a variety of forms and flavours. Some of the well-known FSB products include cheonggukjang of Korea, natto of Japan, and kinema of India or Nepal, which are made with similar recipes [6,7,25]. Alkaline fermentation can break down the soy protein into peptides and amino acids. During the fermentation process, ammonia is generated, resulting in an increase in the pH of the product and the development of an ammonia smell [26,27,28]. The main factors that gave each variety of FSB product its distinct taste, flavour, and texture were the type of local beans used as raw materials, the fermentation processes, the climate, and the production environment.

*Bacillus*, especially *Bacillus subtilis*, has been recognised as the main group of bacteria involved in the fermentation of alkaline-fermented soybean products [6,7,25,26,29,30]. Moreover, this species had previously been isolated from FSBs collected in the Mae Hong Son province of Northern Thailand [31]. However, little is known about the bacterial communities in FSB products, especially the more rare and unique varieties which are produced and consumed by ethnic minorities in remote areas. The *Bacillus* genus was found to be the major group in all of the FSB varieties tested, which supposedly correlates with this genus’ major role in alkaline soybean fermentation. However, the proportions of *Bacillus* in the three FSB types varied. The Lawa FSB had a smaller proportion of this genus than the others, which correlated with the presence of more diverse minor bacterial groups, which might be influenced by the process of production and contamination from the environment.

Interestingly, the minor groups of bacteria found were unique to the FSB from each ethnic group. The presence of some bacterial taxa in the FSBs might be an indicator of poor hygiene conditions, or bacterial contamination from the production environment. In the Karen FSB, *Ignatzschineria* and *Staphylococcus equorum* were found. *Ignatzschineria* is known to be associated with maggots and flies, and some species of *Ignatzschineria* can cause disease in humans [32,33,34]. *Staphylococcus equorum* was found in fermented sausage, in the environment around food processing units, and on surface-ripened Swiss mountain cheese [35,36]. Although the presence of *S. equorum* in soybean products has not been reported, this organism was found in jeotgal (Korean fermented seafood) and can be developed as a starter culture for jeotgal [37]. Moreover, this species was recommended as a starter culture for surface-ripened cheeses, provided that the strain does not have antibiotic resistance [35]. Bacterial network analysis showed no correlation between *Bacillus* and *S. equorum*, but showed a negative correlation between *Bacillus* and *Ignatzschineria*. Considering its common source, it is possible that *Ignatzschineria* might have contaminated the FSB product through flies during the early stages of the fermentation process, but was later overgrown by *Bacillus*. Some members of *Bacillus* are known to produce proteolytic enzymes [38,39] and inhibitory substances, including bacteriocins [40,41], which could aid its utilisation of soy protein and inhibition of *Ignatzschineria*. *Bacillus* could be considered a microbial source of biopreservatives which could also have antagonistic effects on pathogenic and spoilage microbes besides *Ignatzschineria*, thus contributing to the longer shelf-life of FSB products.

In the Lawa FSB, *Atopostipes* and *Yaniella* were found as minor bacterial genera, but in significant proportions. *Atopostipes suicloacalis* is the only species in its genus [42], and this bacterium was isolated from swine manure [42,43]. *Yaniella* belongs to Actinobacteria, and members of this genus were found in forest soil and cheeses [44,45,46]. *Atopostipes* and *Yaniella* from the environment could have contaminated this product. However, as negative correlations were detected between *Bacillus* and these two genera according to the network analysis, these bacteria could have been overgrown and/or inhibited by *Bacillus*, and by its proteolytic enzymes and inhibitory substances potentially secreted into the FSB product. Although the safety impact of *Atopostipes* and *Yaniella* in the Lawa FSB is unclear, their presence could indicate inadequate hygiene practices during production and urge hygiene improvement in the fermentation process.

*Vagococcus* was an intriguing bacterium that was only found in the Shan FSB. This genus belongs to lactic acid bacteria. It has been associated with plant and animal sources used as raw materials for fermented foods, and has been isolated from many indigenous fermented foods [47]. A recent study showed the production of fibrinolytic enzymes from *V. carniphilus* and *V. lutrae* [48]. Although this bacterium was not classified at the species level in our work, its presence points to the possibility that the Shan FSB has a similar function to that reported in the above study. It would be an interesting focus of future work to further explore the functional properties of *Vagococcus* species in this fermented product such as their probiotic properties and their ability to produce health-promoting compounds. The presence of *Proteus*, which was generally known to be associated with soil, decomposing animal matter, sewage, and animal digestive tracts, and was also uniquely found in the Shan FSB, could also point to the need to monitor hygiene practices during FSB production.

*Aneurinibacillus migulanus*, previously known as *Brevibacillus brevis* or *Bacillus brevis* [49], was another bacterium that was specifically found in the Shan FSB. This soil bacterial species has various biological roles, one of which is preventing plant pathogenic microorganisms. It produces bacteriocins and some other functional secondary metabolites [50]. According to one study, it can prevent downy mildew and panama diseases [49,51]. This species can also promote plant rhizobacteria which enhance root development [52]. This species may have entered the Shan FSB through the soybeans used as raw materials for FSB production. Furthermore, this species was found to be a potential probiotic [53].

The Lawa FSB had a greater bacterial diversity than the FSBs of other ethnic groups. The boiling of soybeans in the water retained after cooking sticky rice may have provided an additional carbon source for the growth of various types of bacteria present in the Lawa FSB. In addition, the Lawa FSB was kept next to the stove during and after fermentation. The warm temperature around the stove creates an ideal environment for bacterial growth. The sun-drying step in the Karen FSB manufacturing process could also eliminate some bacteria, resulting in a lower bacterial diversity than the Lawa FSB. As for the Shan FSB, the lower bacterial diversity and the absence of hygiene indicators may be indicative of good manufacturing practice. Moreover, the differences in the bacterial compositions of the FSBs might be attributed to the different bacteria on the banana leaves, which can vary depending on their geographical origins. The greatest bacterial diversity found in the Lawa FSB correlated with the results from the alpha diversity analysis. In addition, the beta diversity analysis pointed out differences in the bacterial taxa and quantity among the FSBs of the three ethnic groups. Another factor that might have contributed to these differences is the availability of nutrients. Many nutritional compositions (fat, protein, carbohydrates, fiber, sugar, sodium, iron, calcium, and potassium) in the Lawa FSB were higher than the others. This may have promoted bacterial growth and could be one of the key factors contributing to the greater bacterial diversity observed in the Lawa FSB. On the other hand, the greater bacterial diversity could be related to the higher nutritional values as a result of various bacterial activities.

The functional gene prediction based on the bacterial compositions in the FSBs also showed unique functions predicted for the Lawa FSB. The highly abundant superpathway of glycolysis that is predicted for the Lawa FSB might be related to its production process, in which the water retained after cooking sticky rice is used to cook the soybean. The additional carbon source obtained from this water might increase the glycolysis activity of the bacteria in the Lawa FSB bacterial community. The functional prediction might describe some bacterial metabolic processes that occurred during soybean fermentation. For example, the D-fructuronate degradation that was predicted for all three FSBs is a pathway that promotes the digestion of pectate and pectin through the D-glucuronate and D-galacturonate pathways [54,55]. These pathways were found in some species of *Bacillus* [56], and they might help release more nutrients from the digestion of soybeans. The abundance of the D-fructuronate and D-galacturonate degradation properties predicted was related to the proportions of *Bacillus* in the communities. Moreover, some functions that are associated with health promotion, especially the synthesis of some amino acids (such as valine and isoleucine), are also related to *Bacillus* proportions. A vitamin (biotin) synthesis function was also abundant in the Karen and Shan FSBs. These predicted functions indicated that these FSBs and *Bacillus*, the major bacterial genus present in the FSBs, can be sources of useful enzymes and beneficial metabolites. However, these functions were predicted using the data of bacterial taxa present in the FSBs; therefore, these predicted functions represent the overall functions of all bacteria in the community. To find an association between a function and a certain type of bacteria, it is necessary to isolate pure cultures and analyse some of their important functions, which can be carried out in a future work. These may include some functions that are related to health promotion, such as amino acid and vitamin synthesis, or those possessing biological activities, such as thiazole biosynthesis. This culture-dependent approach will enable validation of some of these predictions for further applications.

## 5. Conclusions

The fermented soybeans (FSBs) of three Lanna ethnic groups in Northern Thailand—Karen, Lawa, and Shan—contained *Bacillus* as a major bacterial group. The profile of minor bacteria was unique for the FSB of each ethnic group, which could be attributable to the geographical environment and the FSB production processes. Some of the minor bacteria, considering their natural habitats, could be linked to inadequate hygiene practices during food production. Some members of the *Bacillus* genus in all FSBs as well as *Vagococcus* in the Shan FSB could potentially be probiotics or beneficial to human health, and the results of the network analysis and functional prediction partly supported this. Thus, these indigenous fermented soybean products of Lanna heritage are potential functional foods, provided hygiene standards in their production processes are imposed in order to warrant their functionality and food security.

## Figures and Tables

**Table 1:** Nutritional compositions of fermented soybean products from three ethnic groups.

Nutritional Composition	Fermented Soybean		
	Karen	Lawa	Shan
Energy (Kcal/100 g)	310.66	291.35	315.43
Energy from Fat (Kcal/100 g)	162.67	193.65	142.56
Fat (g/100 g)	7.55	8.67	6.98
Protein (g/100 g)	16.33	19.23	18.86
Carbohydrate (g/100 g)	7.34	9.68	8.34
Fiber (g/100 g)	10.57	12.69	11.23
Sugar (g/100 g)	0.26	0.52	0.32
Sodium (Na) (mg/100 g)	27.20	65.67	10.77
Iron (Fe) (mg/100 g)	0.11	0.12	0.14
Calcium (Ca) (mg/100 g)	3.36	4.6	3.47
Potassium (K) (mg/100 g)	22.40	54.37	33.45
Ash (g/100 g)	2.37	4.88	2.87
Moisture (g/100 g)	66.40	57.54	30.65
pH	6.43	6.70	6.29
Water activity (a_w_)	0.85	0.83	0.53

## Data Availability

Publicly available datasets were analysed in this study. These data can be found under BioProject accession number PRJNA747069.

## Supplementary Materials

The following supporting information can be downloaded at: https://www.mdpi.com/article/10.3390/microorganisms11030649/s1, Figure S1: Fruchterman–Reingold plot of network analysis, showing interactions among bacterial taxa in the communities found in alkaline-fermented soybean products of three ethnic groups.
