# Supplementary material for: Bacterial Communities in Lanna Fermented Soybeans from Three Different Ethnolinguistic Groups in Northern Thailand
Source: Microorganisms. 2023 Mar 3;11(3):649. doi: 10.3390/microorganisms11030649 (PMC10056180; doi:10.3390/microorganisms11030649)
Supplement: Supplementary file 1 [file microorganisms-11-00649-s001.zip › microorganisms-2214647-supplementary.pdf]

Supplementary Material

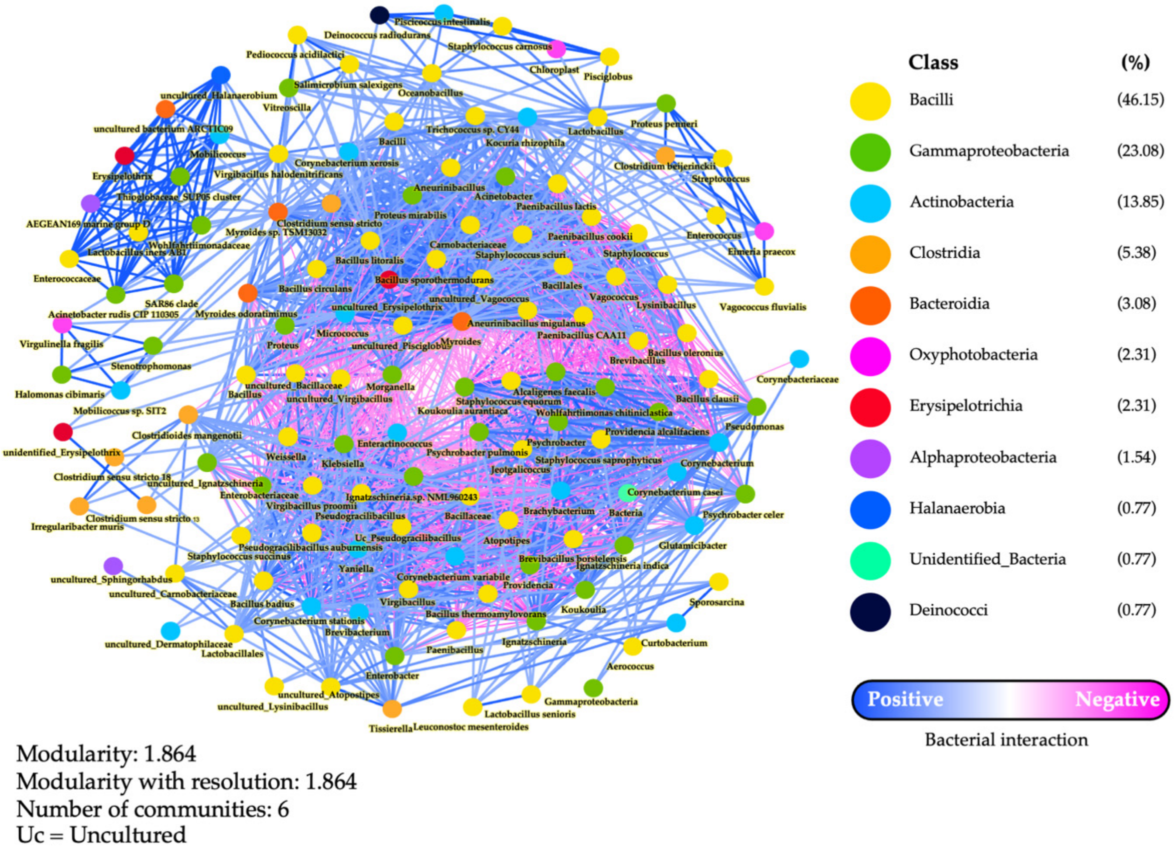

Figure S1. Fruchterman–Reingold plot of network analysis, showing interactions among bacterial taxa in the communities found in alkaline-fermented soybean products of three ethnic groups.
